# A Comparative Study of Cell Culture Conditions during Conversion from Primed to Naive Human Pluripotent Stem Cells

**DOI:** 10.3390/biomedicines10061358

**Published:** 2022-06-09

**Authors:** Irene Romayor, Lara Herrera, Maria Burón, Myriam Martin-Inaraja, Laura Prieto, Jone Etxaniz, Marta Inglés-Ferrándiz, Jose Ramon Pineda, Cristina Eguizabal

**Affiliations:** 1 Cell Therapy, Stem Cells and Tissues Group, Biocruces Bizkaia Health Research Institute, 48903 Barakaldo, Spain; irene.romayorarredondo@osakidetza.eus (I.R.); lara.herreradelval@osakidetza.eus (L.H.); maria.buronaizpiri@donantesdesangre.eus (M.B.); myriam.martininaraja@donantesdesangre.eus (M.M.-I.); laurapril97@gmail.com (L.P.); etxanizjone@gmail.com (J.E.); marta.ingles.ferrandiz@gmail.com (M.I.-F.); 2 Research Unit, Basque Centre for Blood Transfusion and Human Tissues, 48960 Galdakao, Spain; 3 Cell Biology and Histology Department, University of the Basque Country (UPV/EHU), 48940 Leioa, Spain; joseramon.pinedam@ehu.eus; 4 Achucarro Basque Center for Neuroscience, University of the Basque Country (UPV/EHU), 48940 Leioa, Spain

**Keywords:** hiPSCs, hESCs, pluripotency, naive status, primed status, cell differentiation, ectoderm, mesoderm, endoderm

## Abstract

The successful reprogramming of human somatic cells into induced pluripotent stem cells (hiPSCs) represented a turning point in the stem cell research field, owing to their ability to differentiate into any cell type with fewer ethical issues than human embryonic stem cells (hESCs). In mice, PSCs are thought to exist in a naive state, the cell culture equivalent of the immature pre-implantation embryo, whereas in humans, PSCs are in a primed state, which is a more committed pluripotent state than a naive state. Recent studies have focused on capturing a similar cell stage in human cells. Given their earlier developmental stage and therefore lack of cell-of-origin epigenetic memory, these cells would be better candidates for further re-differentiation, use in disease modeling, regenerative medicine and drug discovery. In this study, we used primed hiPSCs and hESCs to evaluate the successful establishment and maintenance of a naive cell stage using three different naive-conversion media, both in the feeder and feeder-free cells conditions. In addition, we compared the directed differentiation capacity of primed and naive cells into the three germ layers and characterized these different cell stages with commonly used pluripotent and lineage-specific markers. Our results show that, in general, naive culture NHSM medium (in both feeder and feeder-free systems) confers greater hiPSCs and hESCs viability and the highest naive pluripotency markers expression. This medium also allows better cell differentiation cells toward endoderm and mesoderm.

## 1. Introduction

In the past two decades, insights into early embryo development have enlarged our perception of pluripotency. Fundamentally, pluripotency is no longer viewed as a fixed state but rather a highly dynamic, malleable signaling network [1]. Mouse embryonic stem cells (mESCs) are one of the earliest and better characterized models of pluripotency [2]. Derived from the inner cell mass (ICM) of mouse blastocysts, mESCs demonstrated characteristic features of pluripotency, including long-term self-renewal, ability to differentiate toward all germ layers, high single-cell clonogenicity and efficient contribution to chimeras [3]. hESCs are derived from human pre-implantation embryos and have an epithelial morphology, could not be propagated efficiently as single cells and have different growth requirements, therefore are markedly different from mESCs [4]. It quickly became evident that hESCs and mESCs relied on different signaling pathways to maintain pluripotency [5]. Some years later, mouse epiblast stem cells (mEpiSCs) were isolated from post-implantation embryos and were found to share many similarities with hESCs. Their transcriptome was similar to that of the post-implantation epiblast [6], indicating that hESCs were more representative of later stages of embryo development. Accordingly, two states of pluripotency were proposed: naive and primed [5]. Therefore, mESCs exist in a naive state, which constitutes the functional in vitro equivalent of the pre-implantation epiblast, while hESCs are in a primed state. The naive state of pluripotency is characterized by an apparently uncompromised differentiation potential, low variability in pluripotency linked gene expression, global DNA hypo-methylation and two active X-chromosomes in female cells. Contrarily, cells in the primed state exhibit distinct pluripotency-associated gene patterns, DNA hyper-methylation and X-chromosome inactivation; this state corresponds to the transition of naive epiblast cells toward a more committed state in vivo [5,6,7].

In 2007, Prof. Shinya Yamanaka reprogrammed adult human dermal fibroblast cells with the introduction of four transcription factors (OCT3/4, SOX2, c-Myc and KLF4) under hESCs culture conditions creating the human induced pluripotent stem cells (hiPSCs) [8]. hiPSCs exhibit discernible gene expression and DNA methylation patterns, some of which could be attributed to their cell type of origin. These epigenetic marks present in hiPSCs may be linked to their primed pluripotency status, which blocks a proper differentiation toward certain cell types depending on the origin of the reprogrammed cell [9,10].

Several groups have studied different strategies to convert hESCs and hiPSCs from primed to naive status. Some of the methods to obtain naive stem cells include the use of different media, such as the naive human stem cell medium (NHSM) [11], t2iLGö medium [12] or the feeder-independent naive embryonic (FINE) medium [13]. These chemical methods to achieve naive status in vitro have drawn the attention of several groups in order to obtain differentiated cells from a more malleable and less committed cell source [14] and to study their cell plasticity potential [15].

In this study, we compare different protocols to convert primed hESCs and hiPSCs into naive status. For this purpose, since some protocols use feeder-free culture conditions and others do it in a co-culture system with feeder layer cells, we report the results obtained from all these different conditions. We studied the effectiveness of the different protocols in terms of pluripotency maintenance, naive markers expression, and differentiation capacity into the three germinal layers. Considering these parameters, we report the best cell source, culture conditions and pluripotency status in order to work with the most malleable cells, thereby improving cell differentiation protocols with the final aim to use cell therapy for clinical applications.

## 2. Materials and Methods

### 2.1. Primed hESCs and hiPSCs Culture

Primed (ES-2) hES cell line and primed (KiPS-4F-1) hiPS cell line were obtained from Spanish National Stem Cell Bank (Banco Nacional de Líneas Celulares, Instituto de Salud Carlos III). Primed cell lines were cultured in feeder conditions using irradiated HFF1 cells and in feeder-free conditions using vitronectin (Life Technologies, Carlsbad, CA, USA) coating. For feeder condition, hESC medium was used [16], with the following composition: KO-DMEM (Life Technologies) supplemented with 20% of KSR (Life Technologies), 1% NEAA (Life Technologies), 1% glutaMAX (Life Technologies), 0.5% Pen/Strep (Life Technologies), 50 μM β-mercaptoethanol (Life Technologies) and 10 ng/mL FGF2 (Miltenyi Biotec, NRW, Germany). Cells were mechanically passaged in clumps once a week. For feeder-free conditions, Essential 8TM medium (E8, Life Technologies) was used. Cells were mechanically passaged in clumps twice a week.

For further experiments, such as qRT-PCR or immunofluorescence, cells were manually picked up. For flow cytometry experiments, feeder conditions hESCs and hiPSCs were manually picked up and fully disaggregated with 0.05% trypsin (Life Technologies) incubation for 5 min at 37 °C. For this purpose, feeder-free conditions hESCs and hiPSCs were manually picked up and fully disaggregated with 0.5 mM EDTA (Life Technologies).

### 2.2. Conversion of Primed hESCs and hiPSCs to Naive Status

Different protocols were tested depending on hESCs and hiPSCs culture conditions. For converting hESCs and hiPSCs cultured on feeder condition, NHSM [11] and t2iLGö [12] media were used. NHSM consisted of KO-DMEM (Life Technologies), 18% KSR, 12.5 μg/mL recombinant human insulin (Sigma-Aldrich, St. Louis, MI, USA), 1% glutaMAX, 1% NEAA, 1% Pen/Strep and 0.1 mM β-mercaptoethanol supplemented with 20 ng/mL hLIF (Peprotech, Cranbury, NJ, USA), 8 ng/mL FGF2, 1 ng/mL TGF-β1 (Peprotech), 1 μM PD0325901 (Axon Medchem, Reston, VA, USA), 3 μM CHIR99021 (Axon Medchem), 10 μM SP600125 (Axon Medchem), 2 μM BIRB796 (Axon Medchem), 5 μM Y-27632 (Axon Medchem) and 5 μM Go6983 (Axon Medchem). t2iLGö consisted of DMEM/F12 (Life Technologies), 1% N2 (Life Technologies), 1% B27 (Life Technologies), 1% NEAA, 1% glutaMAX, 0.1 mM β-mercaptoethanol, 50 μg/mL BSA (Sigma-Aldrich) and 0.5% Pen/Strep supplemented with 20 ng/mL hLIF, 1 μM PD0325901, 1 μM CHIR99021 and 5 μM Gö6983. Primed hESCs and hiPSCs were passaged in clumps and seeded onto new feeder cells. Forty-eight hours later, the medium was changed to NHSM or t2iLGö media conditions. When dome-shaped colonies appeared, naive cells were passaged with 0.05% trypsin (Life Technologies) in NHSM condition and with TrypLE Express (Life Technologies) in t2iLGö condition.

For feeder-free culture conditions, NHSM and FINE [13] culture media were used. FINE medium consisted of DMEM/F12 and Neurobasal medium (1:1 ratio, Life Technologies), 1% N2, 1% B27, 1% glutaMAX, 1% NEAA, 0.1 mM β-mercaptoethanol and 62.5 ng/mL BSA supplemented with 0.1 μM dasatinib (Stemcell Technologies, Shanghai, China), 0.1 μM AZD5438 (Tocris, Bristol, UK), 0.1 μM SB590885 (Sigma-Aldrich), 1 μM PD0325901, 10 μM Y-27632, 20 ng/mL hLIF, 20 ng/mL activin A (Peprotech) and 8 ng/mL FGF2. Forty-eight hours later, the medium was changed to NHSM or FINE media conditions. When dome-shaped colonies appeared, NHSM condition cells were passaged with 0.5 mM EDTA, and FINE cultured naive cells were TrypLE Express and passaged to hypoxia condition at day 5 of culture. For further experiments, undifferentiated colonies were selected, avoiding differentiated cells.

**Figure 1:**
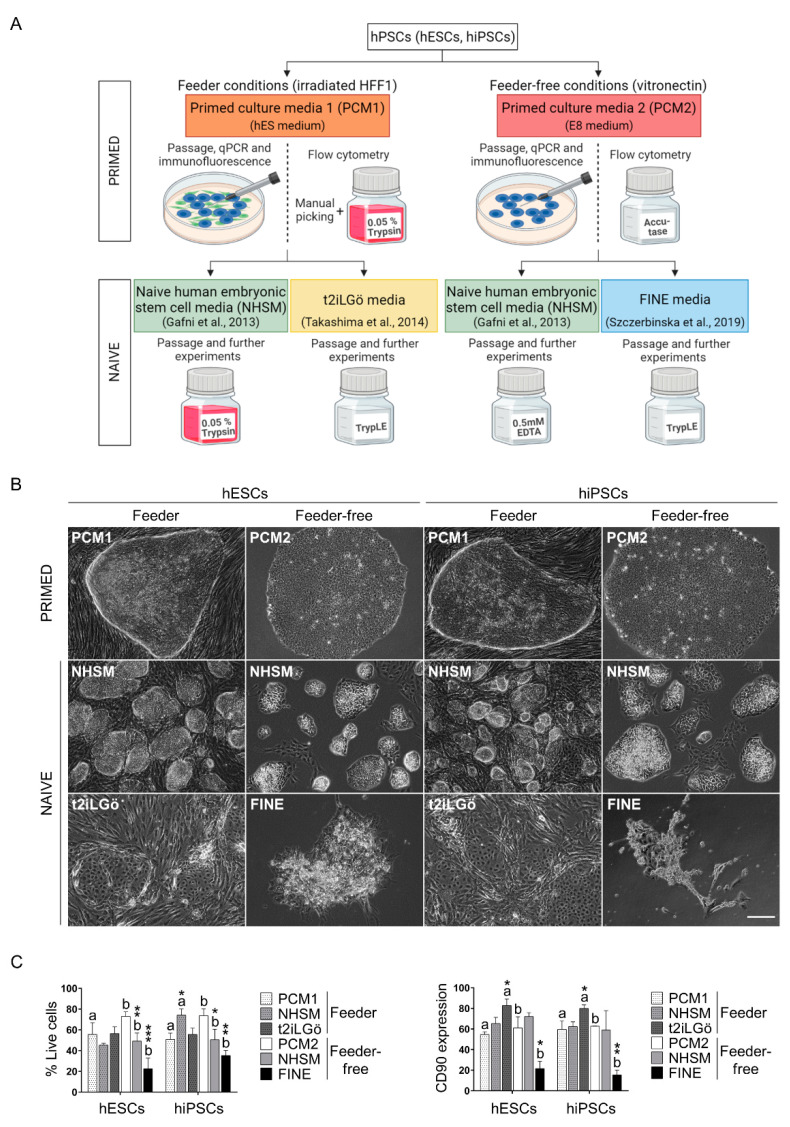
Conversion of primed hPSCs (hESCs, hiPSCs) toward a naive state in different culture media conditions. (**A**) Overview of primed to naive conversion protocol in the feeder and feeder-free culture systems. In feeder conditions, primed hPSCs were maintained in PCM1 (hES medium). Passages and cells used for qRT-PCR and immunofluorescence analysis were mechanically picked up. For flow cytometry assay, cell colonies were disaggregated using 0.05% trypsin following manual picking. Finally, feeder primed hPSCs were cultured in NHSM or t2iLGö media for the primed to naive transition, and cell detachment was performed by 0.05% trypsin and TrypLE solution, respectively. In feeder-free conditions, primed hPSCs were cultured in PCM2 (E8 medium). Colonial cell disaggregation was performed by manual picking in all procedures, except inflow cytometry workflow, in which Accutase solution was used. Lastly, feeder-free primed hPSCs were maintained in NHSM or FINE media for naive conversion, and cells were detached with 0.5 mM EDTA and TrypLE solution, respectively. (**B**) Morphological differences between primed and naive hPSCs colonies in different culture media conditions. Scale bar, 200 µm. (**C**) Flow cytometry analysis of cell viability and CD90 stemness marker expression comparing several culture media conditions. All comparisons are relative to primed media (a* compared to a and b* compared to b. * *p* < 0.1, ** *p* < 0.01 and *** *p* < 0.001). Data are represented as mean ± SD.

Representative drawings of all conversion protocols can be found in Figure 1A.

### 2.3. Flow Cytometry Analysis

The viability of disaggregated primed and naive hESCs and hiPSCs was analyzed with a Life/Death Fixable kit (Invitrogen, Waltham, MA, USA) in a FACS Canto II (BD Biosciences, Franklin Lakes, NJ, USA). Cells were stained with anti-CD24 PE (BD Biosciences, clone ML5) and anti-CD57 APC (BD Bioscience, clone NK-1) primed markers with anti-CD75 FITC (BD Biosciences, clone LN1) and anti-CD130 BV421 (BD Biosciences, clone AM64) naive markers and anti-CD90 PE-Cy7 (BD Biosciences, clone 5E10) stemness marker. Approximately 30,000–50,000 events were acquired for analysis. Populations were analyzed using FlowJo v.X.0.7 (TreeStar Inc., Ashland, OR, USA). See Supplementary Table S1.

### 2.4. Directed Differentiation towards the Three Germ Layers

Endoderm, mesoderm and ectoderm directed differentiation was performed by using STEMdiff™ Trilineage Differentiation Kit (Stemcell Technologies), following the manufacturer’s instructions. Briefly, cells were passaged and cultured in the corresponding medium. Forty-eight hours later, the medium was aspirated and changed for the endoderm, mesoderm or ectoderm medium, respectively. The medium was changed every day until day 5 in the case of endoderm and mesoderm and day 7 for ectoderm. Differentiated cells were ready for the different analyses.

### 2.5. Immunofluorescence and Imaging

hESCs and hiPSCs colonies were seeded on chamber slides (Thermo Fisher Scientific, Waltham, MA, USA) before the staining. Cell colonies were fixed with 4% of formaldehyde (FA) for 20 min at room temperature (RT) and washed 3 times with PBS for 5 min. Cells were permeabilized and blocked using 1X Tris-Buffered Saline (TBS, 50 mM Tris-Cl, pH 7.6; 150 mM NaCl) with 0.5% Triton X-100 (Sigma-Aldrich, 9002-93-1) and 6% Donkey Serum (Millipore, Burlington, MA, USA), S30- 100 ML) for 30 min at RT. Primary antibodies were incubated overnight (OCT4, NANOG, TRA-1-81, SSEA4 and SOX2) or for 48 h (FOXA2, ASMA, SOX9, TUJ1 and GFAP) at 4 °C. After three washes with 1X TBS, corresponding secondary antibodies were added for 2 h at 37 °C (see Supplementary Table S1). Nuclei were counterstained with 0.1 µg/mL DAPI (LifeTechnologies, D21490). Images were taken with a 20× objective of the Leica Microsystems DMi8 inverted microscope.

### 2.6. RNA Extraction and Quantitative Real-Time Polymerase Chain Reaction (qRT-PCR)

Total RNA from cultured hESCs and hiPSCs was extracted and purified using RNAqueous™-Micro Kit (Invitrogen), and its concentration was determined by a Spectro-photometer (NanoDrop ND-1000). cDNA was synthetized utilizing Super-script™ VILO™ cDNA Synthesis Kit (Invitrogen). All samples were analyzed in triplicates, and each reaction included 9 µL master mix and 10 ng cDNA template with a final reaction volume of 10 µL. The master mix comprised gene-specific primers and iTaq Universal SYBR Green Supermix (BIO-RAD, Hercules, CA, USA). Naive status-related genes (*REX1*, *KLF2*, *KLF4*, *KLF17* and *DPPA3*), pluripotency-associated genes (*SSEA4*, *NANOG*, *SOX2* and *OCT4*), and germ layer-specific markers for endoderm (*GATA4* and *GATA6*), mesoderm (*T-BXT* and *HAND1*) and ectoderm (*NESTIN* and *PAX6*) were evaluated. All primers sequences and annealing temperatures are listed in Supplementary Table S2. qRT-PCR gene expression analysis was performed using the CFX96 Detection System (BIO-RAD) in the following conditions: a hot start at 95 °C for 2 min followed by 40 cycles (denaturation at 95 °C for 30 s, annealing for 1 min and elongation at 72 °C for 30 s) and finalized by elongation at 72 °C for 2 min. Data were analyzed utilizing the CFX Manager software according to 2^−ΔΔCt^ method [17], and values were normalized to the housekeeping gene (*GAPDH*).

### 2.7. Statistical Analysis

Experiments were performed in triplicates. Data were analyzed using GraphPad Prism statistical software (version 6.0) and MS Excel. Significance was assessed using the Student’s two-tailed unpaired *t*-test. The criterion for significance was *p* ≤ 0.1. Data are expressed as the mean ± standard deviation (SD).

## 3. Results

### 3.1. Converted hESCs and hiPSCs Present Naive Morphology and Markers

To study and compare the features of naive stem cells, we treated primed hESCs and hiPSCs (cultured in pluripotent culture media -PCM 1 and 2-) using different culture conditions resulting in the combination of (1) three different media (NHSM, t2iLGö and FINE) and (2) the co-culturing of primed cells with a monolayer of feeder cells (NHSM and t2iLGö in feeder co-culture conditions, while NHSM and FINE in feeder-free conditions) (Figure 1A). For better comprehension, we will comment separately on the results obtained regarding the presence/absence of feeder cells.

**Figure 2:**
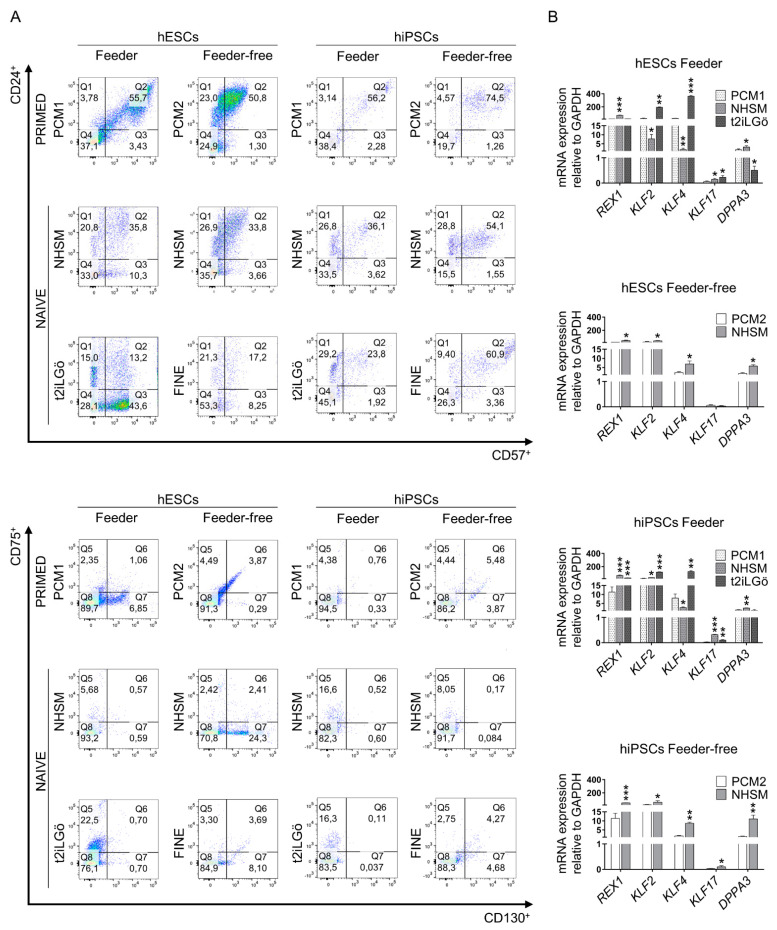
Evaluation of naive conversion of primed hPSCs (hESCs, hiPSCs) in different culture media conditions. (**A**) Flow cytometry analysis of primed status specific markers (CD24 and CD57) and naive status specific markers (CD75 and CD130) on feeder and feeder-free hPSCs cultures. (**B**) Gene expression analysis for naive identity specific genes (*REX1*, *KLF2*, *KLF4*, *KLF17* and *DPPA3*) on feeder and feeder-free hPSCs cultures. All comparisons are relative to primed media (* *p* < 0.1, ** *p* < 0.01 and *** *p* < 0.001). Data are represented as mean ± SD.

Regarding feeder co-culture, we obtained dome-shaped colonies with NHSM at day 5–7 of culture and flat and easily distinguishing colonies with t2iLGö culture medium at day 5–7 of culture (Figure 1B). Viability is maintained along conversion, with a slight decrease in naive hESCs with NHSM medium and a slight increase in naive hiPSCs with NHSM medium (Figure 1C). CD90 stemness marker is significantly higher in t2iLGö culture condition with both pluripotent stem cells (PSC) lines (1.5- and 1.3-fold higher in hESCs and hiPSCs, respectively) compared to their primed counterparts (Figure 1C). When studying primed and naive surface membrane markers, we observed a decrease of primed markers (CD24 and CD57) in both conditions in comparison with the primed control, an increase in naive markers (CD75 and CD130), and, more remarkably, an increase of CD75 marker in t2iLGö culture condition with both PSC lines (Figure 2A). Naive gene expression analysis using naive identity specific genes (*REX1*, *KLF2*, *KLF4*, *KLF17* and *DPPA3*) shows that naive conversion has properly occurred, with their expression in naive conditions significantly higher than in primed conditions. *REX1*, *KLF2* and *KLF17* genes are highly expressed in naive hiPSCs in both conditions, while the *KLF4* gene is highly expressed in naive hESCs in both conditions. *KLF2* and *KLF4* genes are significantly higher expressed in feeder t2iLGö- than in NHSM-naive, while *REX1* and *DPPA3* gene expression is significantly higher in feeder NHSM- than in t2iLGö-naive cells (Figure 2B).

Regarding feeder-free culture condition, we obtained dome-shaped colonies in NHSM at day 5–7 of culture and more disorganized colonies in FINE condition at day 3 of culture (Figure 1B). Viability significantly decreases in both NHSM and FINE conditions with both PSC lines, being more evident in FINE culture condition (Figure 1C). As a matter of fact, due to the low viability of FINE medium, there were not enough cells to perform other experiments apart from flow cytometry. CD90 expression is maintained in NHSM, but its expression in FINE-cultured cells is significantly decreased (Figure 1C). Flow cytometry analysis shows a decrease in primed markers in both PSC lines and culturing conditions, being lower in hESCs than in hiPSCs conversion. On the contrary, naive markers are more expressed in NHSM condition in hiPSCs, while the rest of the conditions are maintained (Figure 2A). Naive gene expression confirms that cells have been correctly converted from primed to naive status, as the expression in naive conditions is significantly higher than in primed conditions. Significance is higher in naive hiPSCs than in naive hESCs in all genes, with the exception of *KLF2* (Figure 2B).

### 3.2. Pluripotency Evaluation of the Naive hESCs and hiPSCs

Subsequently, we assessed the pluripotency status by performing immunofluorescence and qRT-PCR to detect several pluripotency markers in all human PSC lines in both primed and naive statuses.

**Figure 3:**
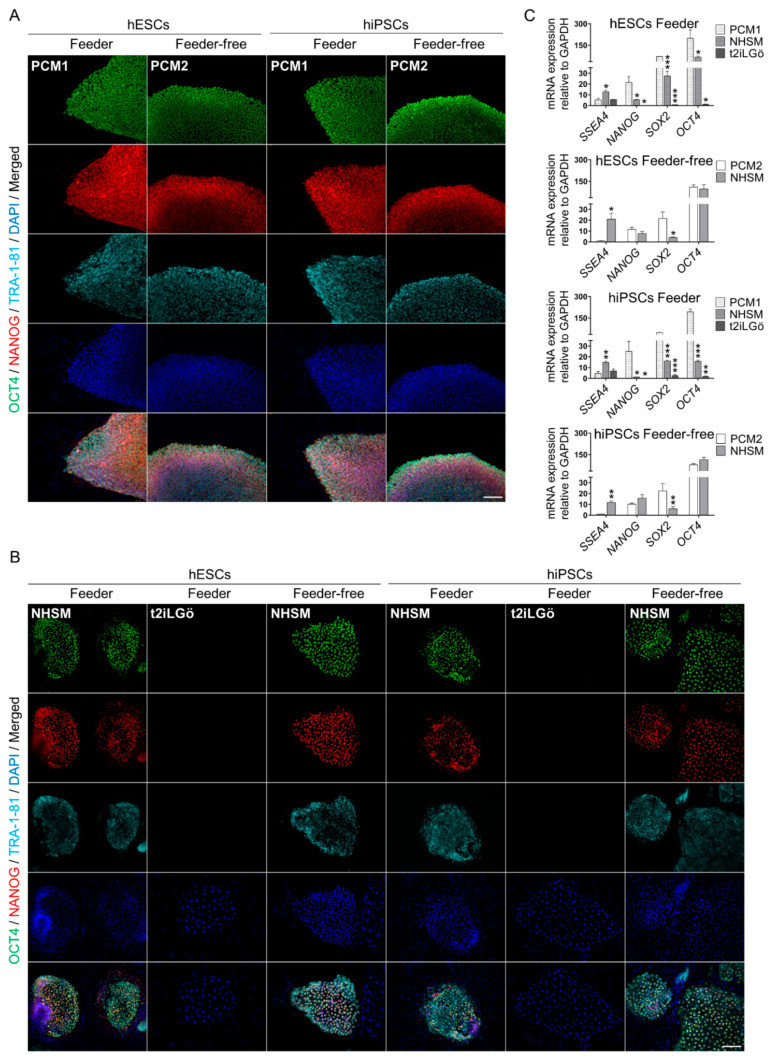
Assessment of primed and naive hPSCs (hESCs, hiPSCs) pluripotency in different culture media conditions. (**A**,**B**) Immunofluorescence analysis of pluripotent capacity specific markers (OCT4, NANOG and TRA-1-81) on primed and naive hPSCs on feeder and feeder-free cultures. Scale bar, 100 µm. (**C**) Gene expression analysis for pluripotency specific genes (*SSEA4*, *NANOG*, *SOX2* and *OCT4*) on feeder and feeder-free hPSCs cultures. All comparisons are relative to primed media (* *p* < 0.1, ** *p* < 0.01 and *** *p* < 0.001). Data are represented as mean ± SD.

Firstly, we observed that primed hESCs and hiPSCs lines in feeder and feeder-free systems were positive for all pluripotent markers studied (OCT4, NANOG and TRA-1-81, Figure 3A,B; SSEA4 and SOX2, Supplementary Figure S1). Moreover, NHSM-naive hESCs and hiPSCs lines were positive for pluripotent markers in both feeder and feeder-free systems, whereas in t2iLGö-naive PSC lines, we found no detection of those pluripotent markers.

Secondly, we analyzed the pluripotency expression genes (*SSEA4*, *NANOG*, *SOX2* and *OCT4*; Figure 3C) in PSC lines in feeder co-culture condition. The *SSEA4* gene is significantly expressed in NHSM-naive hESCs and significantly higher expressed in hiPSCs both in comparison with t2iLGö-naive or primed cells. *NANOG* gene is more expressed in primed than both naive media conversion cells. *SOX2* and *OCT4* genes are more expressed in primed than media conversion naive cells. In general, similar pluripotency expression gene results were observed in hESCs and hiPSCs.

Next, we examined the pluripotency expression genes in PSC lines in feeder-free condition. *SSEA4* gene is significantly higher expressed in NHSM-naive hESCs and significantly higher expressed in both PSC lines, especially in hESCs, in comparison with primed cells. *NANOG* and *OCT4* gene expression are similar, and there are no differences among naive and primed PSC lines. *SOX2* gene is significantly downregulated in NHSM-naive PSC lines and is especially significantly downregulated in hESCs, in comparison with primed cells.

### 3.3. Naive hESCs and hiPSCs Differentiate Readily into All Three Primary Germ Layers

Once we tested the naive cells’ identity and pluripotency in several culture conditions, we aimed to evaluate the lineage-specific differentiation capability of naive hESCs and hiPSCs lines compared to their primed counterparts. For this purpose, primed and naive cells maintained in feeder or feeder-free systems were incubated with endoderm, mesoderm and ectoderm defined media. Then, diverse specific markers for each germ layer were analyzed by immunofluorescence and qRT-PCR.

**Figure 4:**
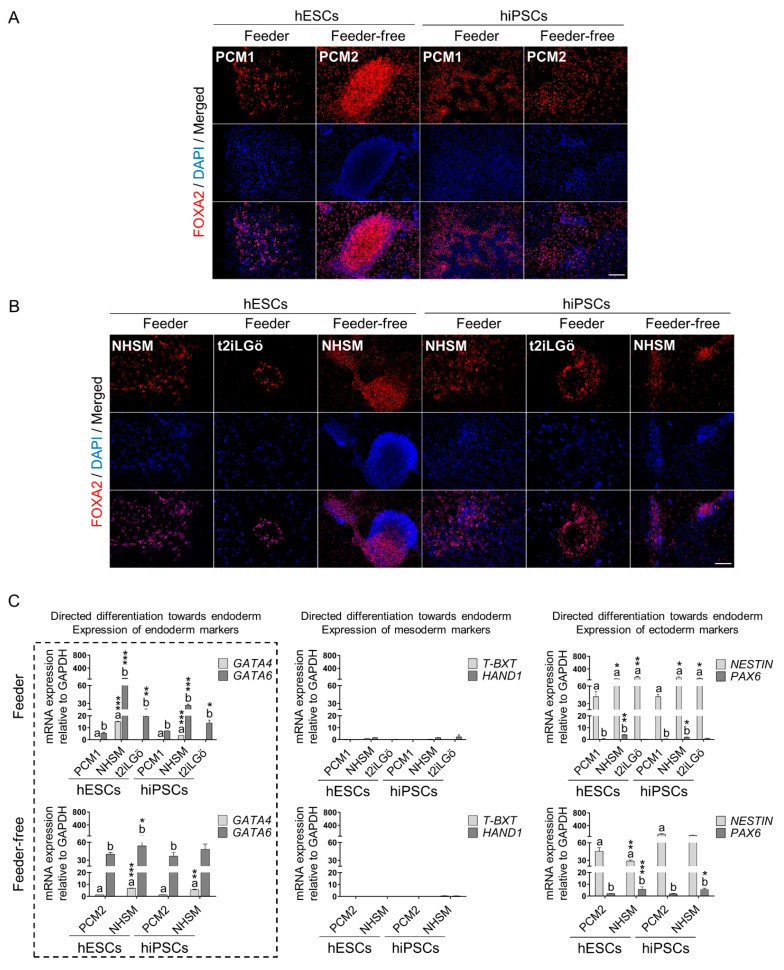
Endoderm layer directed differentiation of primed and naive hPSCs (hESCs, hiPSCs). (**A**,**B**) Immunofluorescence analysis of endoderm specific marker (FOXA2) on primed and naive hPSCs on feeder and feeder-free cultures. Scale bar, 100 µm. (**C**) Gene expression analysis for endoderm specific genes (*GATA4* and *GATA6*) on feeder and feeder-free hPSCs cultures. Specific genes for mesoderm (*T-BXT* and *HAND1*) and ectoderm (*NESTIN* and *PAX6*) were also analyzed in order to determine the lineage heterogeneity in the differentiation procedure. The dashed box represents the gene expression for endoderm-specific markers. All comparisons are relative to primed media (a* compared to a and b* compared to b. * *p* < 0.1, ** *p* < 0.01 and *** *p* < 0.001). Data are represented as mean ± SD.

Firstly, primed and naive hESCs and hiPSCs were specifically directed to differentiate into endoderm. Next, the expression of *FOXA2*, *GATA4* and *GATA6* were examined to corroborate the efficiency of the differentiation protocol. In addition, mesoderm (*T-BXT* and *HAND1*) and ectoderm (*NESTIN* and *PAX6*) biomarkers were assessed in order to determine the heterogeneity of the culture during the differentiation process. Concerning feeder culture conditions, we found evidence of FOXA2 nuclear staining in primed cells as well as in naive cells exposed to both NHSM and t2iLGö media (Figure 4A,B). Furthermore, as shown in Figure 4C, RT-qPCR results revealed that the *GATA4* gene was only expressed by NHSM-naive cells, especially hESCs. In addition, *GATA6* gene levels were significantly upregulated by 16- and 3.6-fold in NHSM-naive hESCs and hiPSCs, respectively, compared to primed ones. In a similar manner, t2iLGö-naive cells also overexpressed the *GATA6* gene with respect to primed cells, showing a 3.6- and 1.9-fold increase in hESCs and hiPSCs, correspondingly (Figure 4C). Regarding feeder-free culture conditions, both primed and NHSM-naive cells manifested intense FOXA2 nuclear staining, similar to cells grown in feeder systems (Figure 4A,B). As observed in Figure 4C, further gene expression analysis of endoderm-specific markers confirmed efficient differentiation. In line with this, results exhibited that NHSM-naive cells significantly overexpressed *GATA4* and *GATA6* genes by around 5- and 1.3-fold, respectively, in contrast to primed cells. Such findings agreed with what we observed before in primed and naive cells cultured on feeder layers, suggesting that naive cells displayed higher potential to differentiate toward the endoderm layer. In relation to the heterogeneity of the endoderm-differentiated cultures, mesoderm-specific markers were detected in neither feeder nor feeder-free conditions. However, we noticed increased levels of ectoderm gene *NESTIN*, principally in feeder naive cells and in feeder-free primed cells. In addition, even though in low levels, ectoderm marker *PAX6* was also uncovered, basically in NHSM-naive cells.

**Figure 5:**
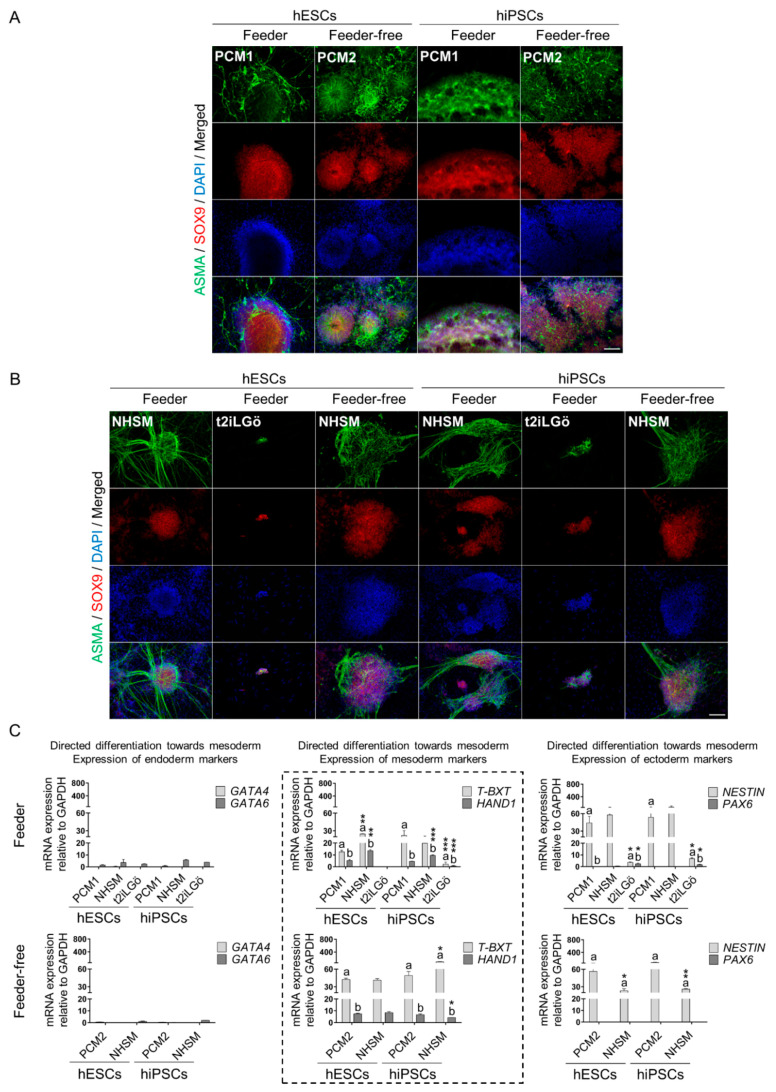
Mesoderm layer directed differentiation of primed and naive hPSCs (hESCs, hiPSCs). (**A**,**B**) Immunofluorescence analysis of mesoderm specific markers (ASMA and SOX9) on primed and naive hPSCs on feeder and feeder-free cultures. Scale bar, 100 µm. (**C**) Gene expression analysis for mesoderm specific genes (*T-BXT* and *HAND1*) on feeder and feeder-free hPSCs cultures. Specific genes for endoderm (*GATA4* and *GATA6*) and ectoderm (*NESTIN* and *PAX6*) were also analyzed in order to determine the lineage heterogeneity in the differentiation procedure. The dashed box represents the gene expression for mesoderm-specific markers. All comparisons are relative to primed media (a* compared to a and b* compared to b. * *p* < 0.1, ** *p* < 0.01 and *** *p* < 0.001). Data are represented as mean ± SD.

Secondly, primed and naive hESCs and hiPSCs were incubated with a mesoderm-defined medium, followed by the evaluation of ASMA, SOX9, T-BXT and HAND1 expression as lineage-specific markers. Moreover, endoderm and ectoderm biomarkers were analyzed as described above. In view of feeder culture conditions, primed cells differentiated toward mesoderm exhibited high expression of ASMA and SOX9 proteins (Figure 5A). In addition, NHSM-naive cells also showed strong SOX9 nuclear staining and the typical muscle tissue morphology signaled by ASMA. In contrast, such markers were hardly detected in t2iLGö-naive cells (Figure 5B). According to these observations, RT-qPCR data revealed that t2iLGö-naive cells did not express mesoderm genes either, while *T-BXT* and *HAND1* were highly upregulated by approximately 2-fold in NHSM-naive cells compared to the primed ones (Figure 5C). With respect to feeder-free systems, ASMA and SOX9 staining was highly detected in primed and NHSM-naive cells, as previously reported in such cells maintained in feeder cultures (Figure 5A,B). Additionally, gene expression results supported that primed and NHSM-naive cells underwent strong mesoderm-directed differentiation, in agreement with feeder conditions. In this regard, cells expressed high levels of *T-BXT* and *HAND1* genes, as evidenced in Figure 5C. Conforming to RT-qPCR results did not exist differences in mesoderm-specific gene expression between primed and NHSM-naive hESCs. Nevertheless, NHSM-naive hiPSCs showed significantly upregulated *T-BXT* and downregulated *HAND1* by around 2-fold compared to the primed hiPSCs. Apparently, feeder-free primed and NHSM-naive cells exhibited similar mesoderm differentiation capacity. Conversely, feeder NHSM-naive cells clearly differentiated better into mesoderm in comparison to primed cells, especially in the case of hESCs. Lastly, similar to feeder systems, mesoderm-induced cells did not express endoderm markers but continued to express high levels of ectoderm gene *NESTIN*, mainly primed cells.

**Figure 6:**
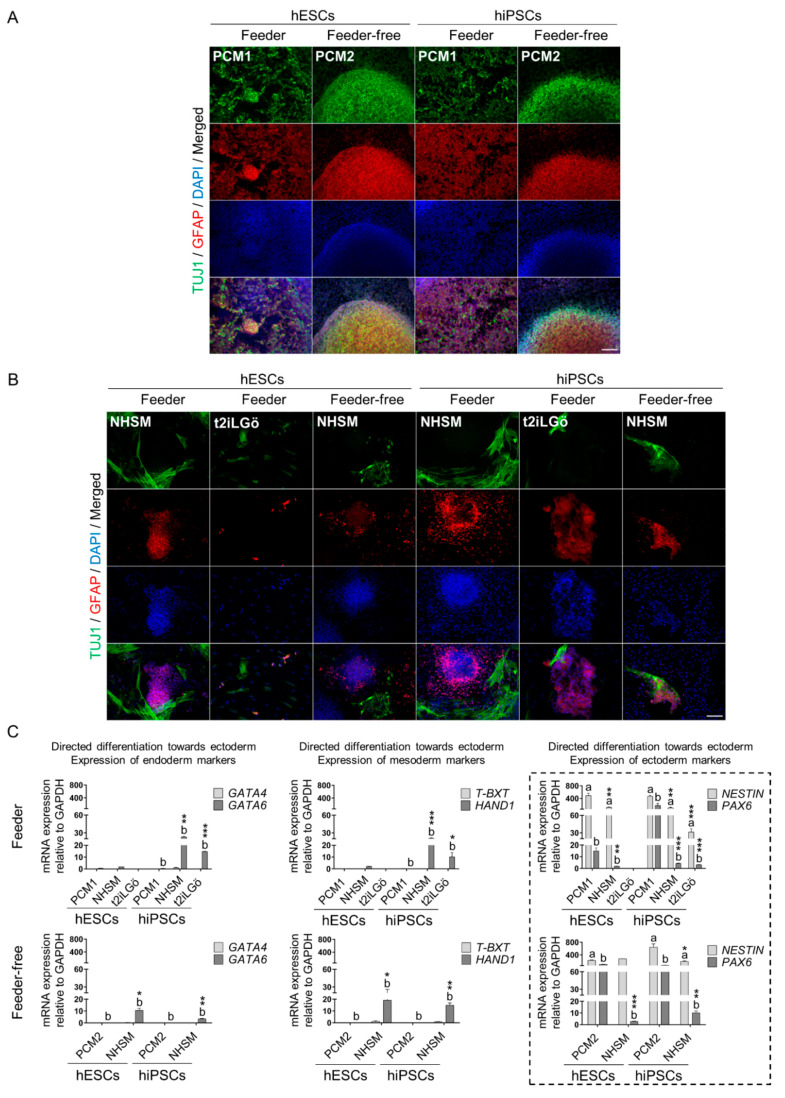
Ectoderm layer directed differentiation of primed and naive hPSCs (hESCs, hiPSCs). (**A**,**B**) Immunofluorescence analysis of ectoderm specific markers (TUJ1 and GFAP) on primed and naive hPSCs on feeder and feeder-free cultures. Scale bar, 100 µm. (**C**) Gene expression analysis for ectoderm specific genes (*NESTIN* and *PAX6*) on feeder and feeder-free hPSCs cultures. Specific genes for endoderm (*GATA4* and *GATA6*) and mesoderm (*T-BXT* and *HAND1*) were also analyzed in order to determine the lineage heterogeneity in the differentiation procedure. The dashed box represents the gene expression for ectoderm-specific markers. All comparisons are relative to primed media (a* compared to a and b* compared to b. * *p* < 0.1, ** *p* < 0.01 and *** *p* < 0.001). Data are represented as mean ± SD.

Finally, primed and naive hESCs and hiPSCs were subjected to ectoderm differentiation, and TUJ1, GFAP, NESTIN and PAX6 expression was determined as germ layer corresponding markers. In addition, the expression level of endoderm and mesoderm genes was also examined, as mentioned before. According to feeder culture conditions, as seen in Figure 6A, primed cells showed TUJ1 and GFAP strong staining. Similarly, as evidenced in Figure 6B, NHSM-naive cells moderately expressed these ectoderm markers. Nonetheless, immunofluorescence images exposed an undoubtedly lower detection of TUJ1 and GFAP proteins in t2iLGö-naive cells, principally in hESCs. Subsequently, as observed in Figure 6C, the expression of *NESTIN* and *PAX6* genes was significantly higher in primed than in naive cells. In detail, primed hESCs exhibited a 4.2- and a 7.8-fold increase in *NESTIN* and *PAX6* expression, respectively, in contrast to NHSM-naive hESCs. In addition, primed hiPSCs showed a 4.7- and a 41.5-fold expansion in these genes compared to NHSM-naive hiPSCs. In the end, t2iLGö-naive cells registered the lowest *NESTIN* and *PAX6* gene expression levels. In fact, t2iLGö-naive hESCs were negative for these biomarkers. Concerning feeder-free culture conditions, both primed and NHSM-naive cells were positive for TUJ1 and GFAP, although the stained area was remarkably lower in naive cells with respect to primed ones, as reported in feeder systems (Figure 6A,B). As observed in Figure 6C, complementary gene expression analyses verified that cells differentiated robustly to ectoderm, being primed cells notably more effective compared to NHSM-naive cells. In line with this, *NESTIN* gene levels showed no differences and a 3.4-fold decrease in NHSM-naive hESCs and hiPSCs, respectively, in contrast to their primed equivalents. Likewise, *PAX6* expression significantly diminished by 36- and 7-fold in NHSM-naive hESCs and hiPSCs, accordingly, compared to their primed correlatives. Equally, similar results were obtained in feeder cultures, indicating that primed cells differentiated more efficiently toward ectoderm. Regarding endoderm and mesoderm biomarkers expression, only NHSM-naive cells manifested medium-high levels of *GATA6* (endoderm) and *HAND1* (mesoderm) genes, as noticed in feeder NHSM-naive hiPSCs.

## 4. Discussion

Over the past decade, the possibility of caching the naive status in vitro has been of great interest to the scientific community, as it provides the opportunity for studying cell development in vitro and the tools for the differentiation toward the desired cell type. Among all the protocols described for this purpose [18], we present here the results of a comparative study of the effectiveness of naive conversion with different culture media and the differences in their pluripotency competences between the naive and the original primed stem cell source, also determining the best cell source for converting primed to naive cells. When converting primed hESCs and hiPSCs to naive cells, a change in morphology is observed, in both feeder and feeder-free conditions, shifting from compact flat colonies (primed) to round dome-shaped ones (naive) as previously described [19,20,21]. Interestingly, when using NHSM medium (in both feeder and feeder-free conditions), more round small colonies arise faster compared to t2iLGö and FINE media. Regarding the viability of the cells, primed hESCs and hiPSCs in the feeder condition present lower viable cells in comparison with primed PSCs in the feeder-free condition, due to the technique used to disaggregate the cells, as previous data reported that the manual picking is more detrimental to the viability of the cells than enzymatic reagents [22]. Besides that, FINE medium cultured naive cells have the lowest viability, being incapable of getting enough cells to perform further experiments. Several groups have described that surface membrane markers’ presence tends to follow the naive conversion, as CD24 and CD57 primed markers get downregulated, and CD75 and CD130 markers get upregulated [23,24]. CD75 marker has a higher expression in t2iLGö culture condition, as the conversion may be quicker, compared to the other methods. Moreover, it has been described that primed marker expression in t2iLGö decreases drastically, [25], being the lowest of all media and lower in t2iLGö naive cells from hESCs. In addition, not only surface markers were analyzed, but naive specific gene expression (*REX1*, *KLF2/4/17* and *DPPA3)* were as well [26,27]. Overall, the gene expression pattern in NHSC in feeder and feeder-free conditions and in t2iLGö culture conditions show cells have reached naive status. *REX1*, *KLF2*, *KLF4* and *KLF17* genes have been reported to be upregulated in naive status [26,28,29], becoming more upregulated in naive hiPSCs, with the exception of *KLF4* being more expressed in naive hESCs. Some of the genes are significantly more expressed in t2iLGö naive cells, as previously reported [30], than in feeder NHSM-naive cells and vice versa. Conversion toward naive cells might behave differently depending on the medium used, as the *KLF4* gene has been described to be crucial for maintaining t2iLGö naive status [12]. Despite the fact that NHSM has been mostly studied in feeder conditions [31], we proved that it is also valid in feeder-free conditions, as all studied naive genes get upregulated, being significantly higher in naive cells from hiPSCs, conferring a more solid naive status than to these cells.

Regarding the human pluripotency status of all PSC lines, it has been described that the standard markers and genes characterizing the pluripotent state, such as *OCT4*, *SOX2*, *NANOG*, *SSEA4* and *TRA-1-81*, are expressed in both naive and primed states [24,32]. The majority have observed that *SSEA4* is expressed in both pluripotency states [11,16,33,34,35,36,37], but one report addresses that *SSEA4* is lower in naive 5i cells defining the human ground state [38]. Despite the fact that all of the hPSC lines derived worldwide share the expression of characteristic pluripotency markers, many differences are arising between lines [39], as we observed differences in pluripotent gene expression between several hPSC lines [16]. This indicates the difficulty of such experimental comparisons requesting a larger number of independent experimental samples that can be simply affected by cell culture conditions, handling skills, methodology of the study and the quality of the hPSC lines utilized [40]. We think that due to this, we were not able to observe any pluripotent markers in t2iLGö culture conditions in both hESC and hiPSC lines or a certain variability in pluripotent gene expression among the different culture conditions (feeder and feeder-free systems) in both naive and primed statuses.

In taking into account our experiments concerning specific differentiation outcomes, our findings demonstrate that primed hESCs and hiPSCs cultured in the feeder and feeder-free systems are able to easily differentiate into the three embryonic germ layers, as diverse studies have reported similar observations [41,42,43,44,45] representing a distinctive feature that defines stemness and pluripotency properties [46]. Thus, together with our analyses of the expression of CD90 and pluripotency-related markers, these results corroborate that primed hESCs and hiPSCs maintain their pluripotent stem cell identity despite the culture conditions tested. As well as primed cells, naive hESCs and hiPSCs can also be directed to differentiate into the three primary germ layers, as previously evidenced [11,12,31,47,48,49]. However, similar to preceding works [31,49,50], we observe that the efficiency of the process greatly diverges between the different culture conditions, cell types and naive resetting media used.

On the one hand, NHSM-naive cells grown with and without feeder layer culture conditions display an elevated capacity to differentiate toward the endodermal lineage, as has been shown before [11]. In fact, NHSM-naive cells exhibit a higher endoderm differentiation capability compared to primed cells, especially feeder hESCs. In line with this, other research groups have described analogous results to ours using hESC line UGent11-70 maintained on feeder conditions [31]. On the other hand, t2iLGö-naive cells also show better differentiation ability into endodermal derivatives with respect to primed ones. Correspondingly, prior works have revealed efficient induction of primitive and mature endoderm-specific markers in t2iLGö-naive hESCs and hiPSCs in vitro [50,51] as we obtain. Actually, a report suggests that hESCs cultured with t2iLGö-modified medium (t2iLGöY) increase their differentiation potential toward endoderm, in contrast to their primed origins [52]. Nevertheless, our results manifest that the effectiveness of the process is notably lower in t2iLGö- as opposed to NHSM-naive cells. In this regard, another research group observed that embryoid bodies (EB) derived from t2iLGöY-naive hiPSCs did not express endoderm biomarkers. On the contrary, those EBs obtained from NHSM-naive hiPSCs show endoderm, mesoderm and ectoderm genes, along with a high propensity to generate teratomas containing tissues representing the three germ layers. This enhanced pluripotency plasticity reported in NHSM-naive cells could be because of a differential expression pattern of naive-associated genes in comparison to t2iLGöY-naive cells. What is more, transcriptome evaluation based on naive and pluripotency markers shows that NHSM-naive cells cluster closer to primed cells than t2iLGöY ones [49], as we notice after evaluating hallmarks related to a pluripotent state. Thus, it seems that NHSM-naive cells conform to an intermediate population between primed and t2iLGöY-naive cells [49,50,51,52,53]. In relation to this, another report suggests that such discrepancies could be justified by the fact that NHSM- and t2iLGö-naive cells comprise different phases of pluripotency [35], even though both are considered stem cells, as they are positive for CD90 marker.

According to such conclusions regarding NHSM naive conversion medium [11,12,31,47,48,49], we also detect that not only endoderm but also mesoderm differentiation capability is amplified in NHSM-naive cells, specifically in cells cultured in feeder systems. Compared to their primed equivalents, NHSM-naive cells significantly overexpressed mesoderm genes after lineage-specific induction, mainly hESCs. Nonetheless, NHSM-naive cells maintained in feeder-free cultures do not exhibit remarkable improvements in mesoderm differentiation competency respecting primed cells. This before-mentioned variability could be explained by the differences in the expression of *OCT4*. Albeit analyzing other naive media, high levels of *OCT4* induce the proliferation of naive cells, while low *OCT4* expression boosts mesoderm differentiation [48]. Similarly, we observed that feeder-free NHSM-naive cells conserve increased *OCT4* expression, describing a self-renewing population. In addition, feeder NHSM-naive cells show downregulated *OCT4* as compared to primed cells, resulting in an increment of their differentiation potential. Contrastingly, t2iLGö-naive cells manifest impaired differentiation ability toward mesodermal derivatives. Such findings coincide with previous studies examining the multi-lineage specification capability of converted naive cells with a t2iLGöY medium. In this regard, the authors propose that a formative transition involving the reduction of naive pluripotency transcription factors in addition to extended differentiation times are necessary to promote the fully developmental qualities of naive cells [54,55,56]. It is tempting to hypothesize that similar capacitation procedures could ameliorate t2iLGö-naive cells productively for mesoderm lineage specification.

Contrary to what we obtained after endoderm and mesoderm differentiation protocols, feeder and feeder-free primed cells display the highest competence for ectoderm definition. Although NHSM-naive cells can be differentiated to ectoderm as well [11,31,35,50,54], ectoderm-specific genes analysis reveals lower process performance in respect to primed cells. In addition, as for the mesoderm layer, t2iLGö-naive cells exhibit a lack of capacity to respond to inductive cues for ectoderm differentiation, particularly hESCs. Altogether, such poor potential for ectoderm definition observed in naive cells could be because of the downregulation of *SOX2* induced by NHSM and t2iLGö media in all culture conditions. *SOX2* plays a critical role in regulating ectoderm differentiation in both hESCs and hiPSCs [57,58]. What is more, a recent report postulates that the low-*SOX2* genotype is related to high self-renewal and increased endoderm and mesoderm differentiation processes in naive hiPSCs [59], coinciding with our observations. Interestingly, SOX2 could interact with OCT4 to modulate differentiation decisions in hESCs in a deeply asymmetric manner. To this concern, reduced SOX2, in contrast to high OCT4 protein levels, promotes mesendodermal and inhibits ectodermal specifications, and vice versa [58], according to our findings. Moreover, SOX2 reduction is more pronounced in t2iLGö- in respect to NHSM-naive cells, leading to a worse differentiation to the ectoderm layer. These dissimilarities support the theory that both NHSM- and t2iLGö-naive cells differ in their pluripotency state [35,50,54], being NHSM-naive cells the most efficient in promoting ectoderm formation. In view of these results, the use of the aforementioned capacitation media [54,55,56] could potentiate such differentiation abilities into ectoderm in naive cells. Finally, gene expression evaluation highlights the upregulation of *NESTIN* in cells differentiated toward endoderm and mesoderm. Such observation is in line with other groups who expose that undifferentiated hiPSCs express high levels of NESTIN, therefore considering an inappropriate ectoderm marker [60]. Thus, TUJ1, GFAP and PAX6 represent more reliable ectoderm biomarkers.

## 5. Conclusions

In the present work, we were able to convert and maintain the pluripotency, stemness and naive status from primed hiPSCs, notably with NHSM and t2iLGö culture conditions. We found that naive culture media (in both feeder and feeder-free systems), especially NHSM, conferred greater hiPSCs and hESCs viability and the highest naive pluripotency markers expression. Based on results from functional tri-lineage differentiation, we conclude that NHSM-naive hESCs cultured in feeder conditions are the best candidates for directed differentiation toward endoderm and mesoderm germ layers. In the case of ectoderm specification, primed hiPSCs in both feeder and feeder-free systems register the most effective developmental potential. The scope of this study is to compare several culture conditions, pluripotent stem cell types and naive conversion media to elucidate the best scenario for the improvement of differentiating protocols toward specific germinal layers.

## Data Availability

Data is contained within the article and supplementary files.

## Supplementary Materials

The following supporting information can be downloaded at: https://www.mdpi.com/article/10.3390/biomedicines10061358/s1, Figure S1: Assessment of primed and naive hiPSCs (hESCs, hiPSCs) pluripotency in different culture media conditions, Table S1: Overview of primary and secondary antibodies, corresponding company, application and dilution of use, Table S2: Overview of genes, primer sequences and annealing temperatures used for RT-qPCR.
