# Supplementary material for: A Comparative Study of Cell Culture Conditions during Conversion from Primed to Naive Human Pluripotent Stem Cells
Source: Biomedicines. 2022 Jun 9;10(6):1358. doi: 10.3390/biomedicines10061358 (PMC9219795; doi:10.3390/biomedicines10061358)
Supplement: Supplementary file 1 [file biomedicines-10-01358-s001.zip › biomedicines-1745556-supplementary.pdf]

Supplementary Materials -biomedicines-1745556

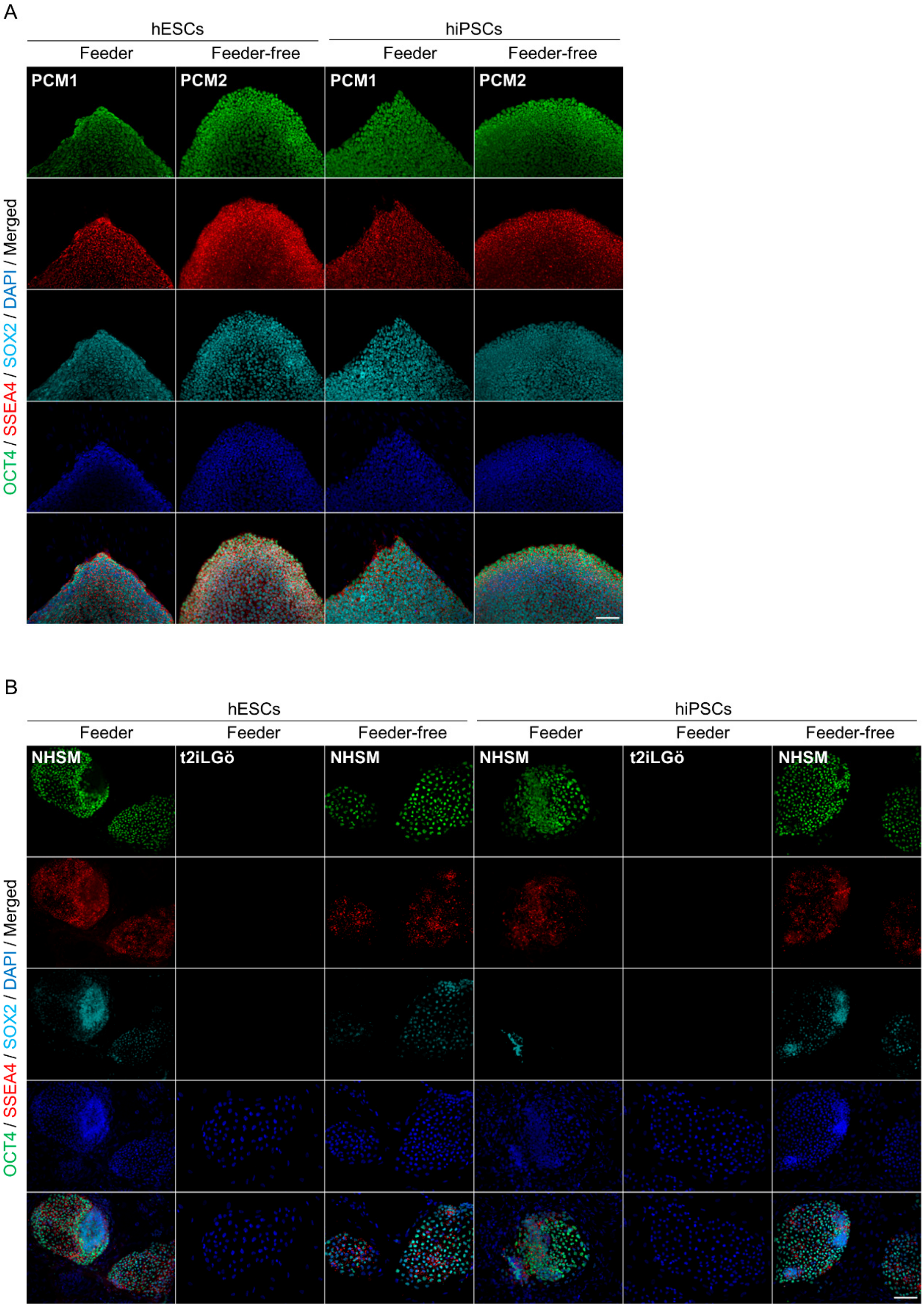

Figure S1: Assessment of primed and naive hiPSCs (hESCs, hiPSCs) pluripotency in different culture media conditions.

Table S1. Overview of primary and secondary antibodies, corresponding company, application and dilution of use.

Antibody	Company (Cat. No.)	Application (Dilution)
Rabbit anti-OCT4	Santa Cruz Biotechnology (sc9081)	IF (1:25)
Mouse anti-SSEA4	Hybridoma Bank (MC-813-70)	IF (1:03)
Goat anti-SOX2	Santa Cruz Biotechnology (sc-17320)	IF (1:25)
Goat anti-Nanog	R&D Systems (AF1997)	IF (1:25)
Mouse anti -TRA-1-81	Millipore (MAB4381)	IF (1:200)
Goat anti-FOXA2	R&D Systems (AF2400)	IF (1:25)
Mouse anti-TUJ1<br>Rabbit anti-GFAP	Covance (MMS-435)<br>Abcam. (ab7260)	IF (1:500)<br>IF (1:1000)
Mouse anti-ASMA<br>Rabbit anti-SOX9	Sigma-Aldrich (A5228)<br>Sigma-Aldrich (AB5535)	IF (1:400)<br>IF (1:300)
Alexa Fluor 488 donkey anti-goat IgG	ThermoFisher Scientific (A11055)	IF (1:200)
Alexa Fluor 546 donkey anti-mouse IgG	ThermoFisher Scientific (A10036)	IF (1:200)
Alexa Fluor 647 donkey anti-goat IgG	ThermoFisher Scientific (A21447)	IF (1:200)
Alexa Fluor 647 donkey anti-mouse IgG	ThermoFisher Scientific (A31571)	IF (1:200)
Alexa Fluor 488 donkey anti-rabbit IgG	ThermoFisher Scientific A21206	IF (1:200)
Alexa Fluor 488 donkey anti-mouse IgG	ThermoFisher Scientific (R37114)	IF (1:200)
Alexa Fluor 546 donkey anti-goat IgG	ThermoFisher Scientific (A11056)	IF (1:200)
Alexa Fluor 546 donkey anti-rabbit IgG	ThermoFisher Scientific (A10040)	IF (1:200)
LIVE/DEAD APC.Cy7 (viability)	Invitrogen (L-34975)	FC (1:1000)
Mouse anti- CD24 PE	BD Bioscience (560991)	FC (1:50)
Mouse anti- CD57 APC	BD Bioscience (560845)	FC (1:50)
Mouse anti- CD75 FITC	BD Bioscience (555654)	FC (1:50)
Mouse anti- CD130 BV421	BD Bioscience (566223)	FC (1:50)
Mouse anti- CD90 PE.Cy7	BD Bioscience (561558)	FC (1:50)

Table S2. Overview of genes, primer sequences and annealing temperatures used for RT-qPCR.

Gene		Primer Sequence (5´-3´)	Annealing temperature (°C)
**SSEA4**	Stage-specific embryonic antigen-4	Fw: TGGACGGGCACAACTTCATC<br>Rv: GGGCAGGTTCTTGGCACTCT	60
**NANOG**	Homeobox protein NANOG	Fw: TCTCCAACATCCTGAACCT<br>Rv: GCGTCACACCATTGCTAT	57
**SOX2**	SRY-Box transcription factor 2	Fw: GCACAACTCGGAGATCAG<br>Rv: CAGCGTGTACTTATCCTTCT	57
**OCT4**	Octamer-binding transcription factor 4/POU5F1	Fw: AGAGGCAACCTGGAGAAT<br>Rv: ATAGTCGCTGCTTGATCG	57
**REX1**	Reduced expression 1	Fw: CCTGCAGGCGGAAATAGAAC<br>Rv: GCACACATAGCCATCACATAAGG	60
**KLF2**	Kruppel-like factor 2	Fw: CACCAAGAGTTCGCATCTGAAGG<br>Rv: TACATGTGCCGTTTCATGTGCAG	57
**KLF17**	Kruppel-like Factor 17	Fw: GGGATGGTGCGATAGATTCA<br>Rv: GCCTCACCCTCACCTAACAA	57

**DPPA3**	Developmental pluripotency associated 3	Fw: ATCGGAAGCTTTACTCCGTCGAG<br>Rv: CCCTTAGGCTCCTTGTTTGTTGG	57
**NESTIN**	Neuroepithelial stem cell protein	Fw: CCTGGGAAAGGGAGAGTACC<br>Rv: TGGTCCTTCTCCACCGTATC	55
**PAX6**	Paired box 6	Fw: GTCCATCTTTGCTTGGGAAA<br>Rv: TAGCCAGGTTGCGAAGAACT	63
**T-BXT**	T-Box Transcription Factor T	Fw: GCAAAAGCTTTCCTTGATGC<br>Rv: ATGAGGATTTGCAGGTGGAC	60
**HAND1**	Heart and neural crest derivatives expressed 1	Fw: CTGGCTCTTTCTCTCTTGTC<br>Rv: CGTCTGGTTCTCTTTCTCAG	60
**GATA4**	GATA binding protein 4	Fw: GGGACGGGTCACTATCTTGTG<br>Rv: GGTGGTGGTCTGGCAGTT	57
**GATA6**	GATA binding protein 6	Fw: GTGCCCAGACCACTTGCTAT<br>Rv: CCCTGAGGCTGTAGGTTGTG	60
**GAPDH**	Glyceraldehyde-3-phosphate dehydrogenase	Fw: GGTCGGAGTCAACGGATTTG<br>Rv: TGGAAGATGGTGATGGGATT	60
